# Evidence for Persistent Heteroplasmy and Ancient Recombination in the Mitochondrial Genomes of the Edible Yellow Chanterelles From Southwestern China and Europe

**DOI:** 10.3389/fmicb.2021.699598

**Published:** 2021-07-14

**Authors:** Ying Zhang, Shaojuan Wang, Haixia Li, Chunli Liu, Fei Mi, Ruirui Wang, Meizi Mo, Jianping Xu

**Affiliations:** ^1^State Key Laboratory for Conservation and Utilization of Bio-Resources in Yunnan, Key Laboratory for Southwest Microbial Diversity of the Ministry of Education, Yunnan University, Kunming, China; ^2^School of Life Sciences, Yunnan University, Kunming, China; ^3^Qicai Yunnan Primary School Affiliated with Yunnan Normal University, Kunming, China; ^4^Kunming Edible Fungi Institute of All-China Federation of Supply and Marketing Cooperatives, Kunming, China; ^5^Research Institute of Nutrition and Food Science, Kunming Medical University, Kunming, China; ^6^Department of Biology, McMaster University, Hamilton, ON, Canada

**Keywords:** DNA barcoding, speciation, biogeography, heteroplasmy, nuclear-mitochondrial incongruence, yellow chanterelles

## Abstract

Mitochondrial genes and genomes have patterns of inheritance that are distinctly different from those of nuclear genes and genomes. In nature, the mitochondrial genomes in eukaryotes are generally considered non-recombining and homoplasmic. If heteroplasmy and recombination exist, they are typically very limited in both space and time. Here we show that mitochondrial heteroplasmy and recombination may not be limited to a specific population nor exit only transiently in the basidiomycete *Cantharellus cibarius* and related species. These edible yellow chanterelles are an ecologically very important group of fungi and among the most prominent wild edible mushrooms in the Northern Hemisphere. At present, very little is known about the genetics and population biology of these fungia cross large geographical distances. Our study here analyzed a total of 363 specimens of edible yellow chanterelles from 24 geographic locations in Yunnan in southwestern China and six geographic locations in five countries in Europe. For each mushroom sample, we obtained the DNA sequences at two genes, one in the nuclear genome and one in the mitochondrial genome. Our analyses of the nuclear gene, translation elongation factor 1-alpha (*tef-1*) and the DNA barcode of *C. cibarius* and related species, suggested these samples belong to four known species and five potential new species. Interestingly, analyses of the mitochondrial ATP synthase subunit 6 (*atp6*) gene fragment revealed evidence of heteroplasmy in two geographic samples in Yunnan and recombination within the two new putative species in Yunnan. Specifically, all four possible haplotypes at two polymorphic nucleotide sites within the mitochondrial *atp6* gene were found distributed across several geographic locations in Yunnan. Furthermore, these four haplotypes were broadly distributed across multiple phylogenetic clades constructed based on nuclear *tef-1* sequences. Our results suggest that heteroplasmy and mitochondrial recombination might have happened repeatedly during the evolution of the yellow chanterelles. Together, our results suggest that the edible yellow chanterelles represent an excellent system from which to study the evolution of mitochondrial-nuclear genome relationships.

## Introduction

Fungi are important components of natural ecosystems. Many fungi also play significant roles in human and animal health, agriculture, biotechnology, and forestry. Some of these fungi grow in a mutually beneficial relationship with the root tips of plants, forming mycorrhizal associations. In these mycorrhizal fungi, their mycelia help plants obtain essential minerals, nitrogen and water from the soil and contribute to the plants’ nutrition, disease resistance and drought tolerance (Brundrett, 2004). In return, plants provide their fungal partners with carbohydrates for their growth and reproduction. In forest ecosystems, one of the most common fungal-plant root associations (called mycorrhiza) involves the Basidiomycetes. Many of these basidiomycete fungi also produce conspicuous fruiting bodies—mushrooms. Some of these mushrooms, such as the chanterelles, are harvested as a source of highly prized food for humans.

Mitochondria are the powerhouse of all eukaryotes, responsible for generating the universal cellular energy currency ATP through oxidative phosphorylation. In fungi, previous studies have shown that mitochondria play many other roles, including aging in eukaryotes (Stojkovic et al., 2017), and virulence (Verma et al., 2018) and resistance to antifungal drugs in fungal pathogens (Vielba-Fernandez et al., 2018). In addition, mitochondrial genomes provide excellent materials for population and evolutionary studies. This is especially true in fungi where diverse patterns of mitochondrial inheritance have been observed, from uniparental to biparental inheritance (Zhou et al., 2010; de la Providencia et al., 2013; Xu and Li, 2015; Fourie et al., 2018; Mendoza et al., 2020). However, aside from a few model organisms, our knowledge about the mechanisms of fungal mitochondrial genomes and their inheritance is very limited (Basse, 2010; Wilson and Xu, 2012; Xu and Wang, 2015). In general, in contrast to the Mendelian inheritance of nuclear genes and genomes, the mitochondrial genes and genomes are inherited uniparentally with a preference for the maternal parent, with little or no evidence of recombination in most fungi, animals, and plants (Birky, 1995). As a consequence, all copies of the mitochondrial DNA (mtDNA) of an individual are typically identical to each other and to one of the parental mitochondrial genomes, a condition known as homoplasmy (Ling et al., 2011). Homoplasmy could be achieved through processes that prevent mtDNA transmission from one of the two parents, including no contribution from one parent due to fertilization mechanisms, targeted elimination of mtDNA from one parent through mitophagy, and/or the restriction-modification system (Shibata and Ling, 2007; Sato and Sato, 2011).

In filamentous fungi, the cells at the junctions of mating where the two parental homokaryons meet may be heteroplasmic, with each mated cell containing mtDNA from both parents [e.g., in the button mushroom *Agaricus bisporus* (Xu et al., 1996)]. In Baker’s yeast *Saccharomyces cerevisiae*, the zygotes are almost universally heteroplasmic. However, in the human yeast pathogen *Cryptococcus neoformans*, heteroplasmy is only found in the zygotes when sex-determining genes are knocked out (Yan and Xu, 2003; Sun et al., 2020). In most instances of observed heteroplasmy, the heteroplasmic cells are typically transient, with rapid segregation of parental mtDNA genotypes into homoplasmic progeny cells to form homoplasmic mycelia and fruiting bodies (Birky, 2001; Barr et al., 2005; Wilson and Xu, 2012; Xu and Wang, 2015). However, while most reported heteroplasmy in fungi were from laboratory crosses, heteroplasmy has also been found in natural strains in a few basidiomycete fungi. For example, in our ongoing investigation of population genetics on edible basidiomycete mushrooms in southwestern China, over 87% of *Thelephora ganbajun* samples showed evidence of heterozygosity in the mitochondrial genes *cox1* or *cox3*, representing the first evidence of stable heteroplasmy in natural populations of basidiomycete fungi (Wang et al., 2017). In addition, we found clear evidences of phylogenetic incompatibility between two mitochondrial genes (*atp6* and *cox3*) among geographic populations of another basidiomycete mushroom, an *Russula virescens* ally (Cao et al., 2013). At present, the dynamics of heteroplasmy and recombination, while critical for advancing our knowledge of mitochondrial evolution, has not been critically investigated (Li et al., 2018).

The basidiomycete *Cantharellus cibarius* and related species are broadly found in the northern hemisphere, from Europe to North America and Asia (Buyck et al., 2014). These mushrooms can form symbiotic ectomycorrhizal relationships with many plants and are often found in mossy coniferous and birch forests. They are among the most prominent wild edible mushrooms in the northern hemisphere. Recently, phylogenetic analyses based on the translation elongation factor 1 (*tef-1*) has identified that the following 21 species *C. altipes*, *C. tenuithrix*, *C. lilacinopruinatus*, *C. ferruginascens*, *C. amethysteus*, *C. lewisii*, *C. lateritius*, *C. confluens*, *C. spectaculus*, *C. roseocanus*, *C. flavus*, *C. phasmatis*, *C. isabellinus*, *C. tomentosus*, *C. avellaneus*, *C. tabernensis*, *C. appalachiensis*, *C. decolorans*, *C. texensis*, *C. cinnabarinus*, were phylogenetically related to *C. cibarius* (Buyck and Hofstetter, 2011; Foltz et al., 2013; Buyck et al., 2014). However, most of these species appear to be geographically specific. For example, *C. cibarius* sensu stricto is only found in Scandinavia and northern Japan (Ogawa et al., 2018). Therefore, the idea that Europe and the warm-temperate and subtropical parts of North America have species of *Cantharellus* in common, has been progressively abandoned. Consequently, it’s increasingly recognized that the use of European species names of *Cantharellus* for those found in North America and Asia is likely incorrect, especially for those closely related to *C. amethysteus* and *C. cibarius* (Redhead et al., 1998; Buyck and Hofstetter, 2011). At present, while updates on *Cantharellus* taxonomy have progressed relatively quickly in western Europe and North America, that in Asia remains very fragmented. However, recent studies have reported several new chanterelles from China (Shao et al., 2011; Tian et al., 2012; Shao et al., 2014, 2016a,b; An et al., 2017; Jian et al., 2020), Japan (Suhara and Kurogi, 2015; Ogawa et al., 2018), Korea (Antonin et al., 2017; Buyck et al., 2020), Malaysia (Eyssartier et al., 2009; Buyck et al., 2014), India (Das et al., 2015; Buyck et al., 2018), and Iran (Parad et al., 2018). Recently, a large-scale survey of wild edible mushrooms in local markets in Yunnan province, southwestern China, revealed that there was at least one cryptic species within this group of species in this region (Zhang et al., 2021). This study analyzed 96 samples using sequence information at both the *tef-1* locus and the internal transcribed spacer (ITS) regions of the ribosomal RNA gene cluster. However, a very conservative cutoff for species delimitation was used in new species estimation in this study (Zhang et al., 2021). Another study identified that most of the edible yellow chanterelles old in the Yunnan local markets belonged to *C. Yunnanensis* based on both morphological and *tef-1*sequences, and not the traditionally assumed *C. cibarius* (Shao et al., 2021). Unfortunately, the rRNA gene cluster often fail to distinguish closely related species in *Cantharellus* due to unreadable sequence chromatographs and/or limited DNA sequence variations. Sequence information from other genes as well as more extensive sampling is needed to obtain a better understanding of the evolutionary history of *Cantharellus* and better insights on dispersal routes and speciation events in the Northern Hemisphere (Olariaga et al., 2017).

Chanterelle is the common name for mushrooms in the genus *Cantharellus* and in other morphologically related genera. The edible yellow chanterelles are among the most popular edible wild mushrooms in the world and include *C. cibarius* and related species. There are specialized companies that commercialize fruit bodies of chanterelles of different sizes and presentations, e.g., as fresh, frozen, dry, or salted, with geographic specifications. Morphologically, species in yellow chanterelles are very similar to each other and all species are harvested as food and some of them are globally traded. The combined global commercial value of yellow chanterelles has been estimated at more than one billion US dollars annually (Watling, 1997; Wang and Hall, 2004). One of the main global centers of production and harvesting of edible yellow chanterelles is Yunnan Province in Southwestern China. Due to its highly variable climate and diverse topography, Southwestern China (mainly Yunnan Province) is recognized as one of the world’s 34 biodiversity hotspots (Myers et al., 2000). Relevant to this study, Yunnan is also one of the world’s most important areas for wild mushroom harvesting and trading. For example, of the 2,000 or so wild edible mushroom species in the world, over 900 are found in Yunnan (Yang, 2002; Feng and Yang, 2018). Based on ITS barcoding, our recent survey identified a high species diversity of wild mushrooms in local markets, including a large number of putative new taxa (Zhang et al., 2021). Some of these mushrooms are very important to both the local and regional economy. Most of these species are ectomycorrhizal, contribute significantly to the health of trees and forest ecosystems. Examples of these economically important ectomycorrhizal mushrooms around the globe include *Boletus edulis, Thelephora ganbajun, Tricholoma matsutake, Russula virescens, Russulavinosa* and *C. cibarius* etc.

In this study, we analyzed the DNA sequence variation in the mitochondrial *atp6* gene fragment for a total of 363 mushroom samples of edible yellow chanterelles. Our analyses found that two nucleotide sites within this gene fragment contained sequence heterogeneity within two individual fruiting bodies and that all four possible genotypes at these two sites were found in several geographic populations from Yunnan. Similarly, three additional heterozygous loci were found in another fruiting body collected in a different geographic population. Interestingly, seven out of all eight possible haplotypes at these three nucleotide sites were found in our samples, consistent with recombination. Finally, the phylogenetic distributions of two polymorphic nucleotide sites (sites 101 and 109) within the *atp6* gene fragment were compared with sequence variation at the DNA barcode *tef-1* for edible yellow chanterelles in Yunnan and Europe.

## Materials and Methods

### Mushroom Sampling, DNA Isolation and Gene Sequencing

In this study, we obtained 219 fruiting bodies with macro-morphological characteristics similar to *C. cibarius* and related species from 24 counties in Yunnan province. The sampled areas in Yunnan spanned about 480 km from east to west and about 550 km from north to south. In addition, we obtained 144 yellow chanterelle samples from local markets in five countries (Finland, France, Hungary, Portugal, and Russia) in Europe. These 363 samples were all included in the study. The geographic coordinates and the sample size from each site are presented in Table 1.

**TABLE 1:** Distribution and diversity of *tef-1* sequence types for yellow chanterelles across southwestern China and Europe.

Geographic population (province)/collecting (city)	Latitude (°N)	Longitude (°W)	sample size	ST(no. of isolates in each ST)
Russia (1)	55.45	37.37	27	12(1) 13(2) 14(2) 16(22)
France (2)	45.28	4.2	15	6(1) 14(4) 16(10)
Portugal (3, 4)	38.3	28	65	14(8) 16(45) 17(3) 37(1) 38(8)
Hungary (5)	47.26	19.15	12	2(1) 8(1) 16(10)
Southern France (6)	41.18	2.06	10	1(1) 3(1) 8(1) 16(7)
Finland (8, 9)	60.13	24.5	15	10(1) 11(1) 14(2) 15(1) 16(10)
Changning (CN)	24.67	102.14	3	4(1) 45(2)
Dali (DL)	25.69	100.19	9	5(8) 45(1)
Fengqing (FQ)	24.58	99.91	5	45(5)
Jiangcheng (JC)	22.58	101.88	12	9(1) 19(1) 27(1) 31(2) 41(1) 45(6)
Jianchuan (JChuan)	25.82	100.55	16	25(1) 28(1) 30(1) 31(5) 32(1) 40(1) 45(6)
Jinning (JN)	23.88	102.58	9	23(1) 25(1) 31(3) 35(2) 45(2)
Kunming (KM)	25.04	102.73	5	22(1) 28(1) 33(1) 34(1) 36(1)
Lancang (LAN)	23.38	100.55	1	5(1)
Lijiang (LJ)	26.88	100.25	8	7(1) 35(3) 39(1) 45(3)
Lincang (LC)	25.21	100.09	9	40(3) 45(6)
Lufeng (LF)	25.15	101.26	1	5(1)
Menghai (MH)	21.96	100.45	20	5(3) 31(1) 40(5) 45(11)
Nanhua (NH)	25.55	101.26	8	23(1) 31(1) 35(5) 47(1)
Nanjian (NJ)	25.04	100.51	1	31(1)
Pu’er (PE)	23.33	100.50	1	29(1)
Shangyun (SY)	22.35	99.98	5	5(5)
Shizong (SZ)	24.83	103.98	21	26(1) 27(6) 28(7) 31(2) 32(1) 35(3) 45(1)
Shuangjiang (SJ)	23.45	99.85	7	5(3) 44(1) 45(3)
Xundian (XD)	24.67	103.25	19	18(2) 21(1) 31(1) 40(1) 45(13) 46(1)
Xiangyun (XY)	25.47	100.56	12	26(1) 31(2) 35(7) 45(1) 46(1)
Yimen (YM)	24.57	101.00	23	18(3) 20(1) 40(2) 43(1) 45(14) 46(1)
Yunxian (YX)	24.44	100.12	2	4(1) 24(1)
Yongping (YP)	24.67	102.14	2	5(2)
Zhongdian (ZD, A)	27.78	100.97	20	7(15) 22(1) 24(1) 35(3)
Total			363	

DNA extraction, PCR, sequencing, and sequence alignment all followed those of Wang et al. (2017). These samples were first analyzed based on their sequences at the *tef-1* gene, the preferred DNA barcode for *Cantharellus* (Buyck and Hofstetter, 2011; Antonin et al., 2017; Olariaga et al., 2017). In our study, DNA sequences at the nuclear*tef-1*gene were used to confirm phylogenetic affiliations of all our specimens (see below). In addition, the mitochondrial *atp6* gene sequences were obtained for 327 specimens. The primer pairs, tef-1-S (5′ ACCAGAACGACGCCGCTCAA 3′) and tef-1-A (5′ TTGTCGCCGTGCCAACCAG 3′), ATP6-S (5′ AACACCGGGTACATTTCT 3′) and ATP6-A (5′ TACTTACGGCGATTTCTA 3′) were used to amplify and sequences the *tef-1* and *atp6* gene fragments, respectively.

### mtDNA Heterozygosity

We observed mtDNA heterozygosity, shown as double peaks at specific nucleotide sites in the chromatograms of the *atp6*gene, in several specimens. We investigated whether the observed mtDNA heterozygosity was due to nuclear mt paralogs (numts) or the presence of more than one allele in the mitogenome within each of these specimens. To distinguish these two possibilities, we used two approaches. In the first, we confirmed the nucleotide sequence similarity between our sequences and that of the sequenced genome of *C. cibarius/*sample MG75 (GenBank number: QOWL00000000.1) through Basic Local Alignment Search Tool (BLAST). [Note: Based on its *tef-1* sequence, sample MG75 likely belongs to *C. tuberculosporus.*] The second approach was through copy number assessment using a technique called absolute quantification PCR (AQ-PCR) (Leong et al., 2007).

**FIGURE 1:**
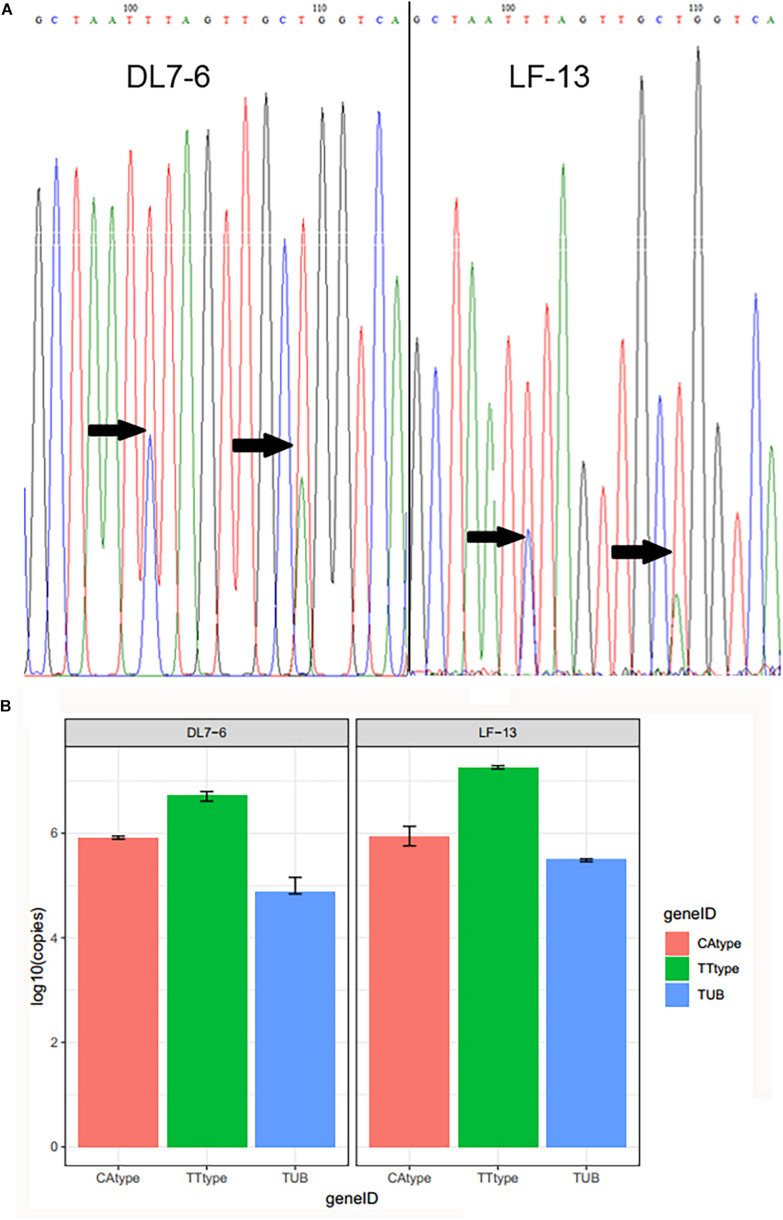
**(A)** Heterozygous sites of mitochondrial gene *atp6* and **(B)** copy numbers of higher peak and lower peak sequences relative to the reference nuclear *tub* gene in samples DL7-6 and LF-13 from Yunnan.

In the second approach, for samples with evidence of heterogeneity at the *atp6* gene, we determined their relative abundances in the fruiting bodies between the two combinations, one combination with peaks (TT) and the second combination with peaks (CA) respectively (Figure 1A) using AQ-PCR. Their abundances were compared with that of a nuclear gene, β-tubulin (*tub*). Since chanterelle mushrooms are diploids, each cell contains two haploid nuclei and multiple mitochondrial genomes. Thus, there would be two copies of each nuclear gene such as *tub* within each cell while multiple copies of mitochondrial genes. In this case, the relative copy numbers of the two *atp6* alleles can be compared with the copy number of *tub* to determine within each sample whether the *atp6* alleles are located in the nuclear genome or the mitochondrial genome. Our specific protocols are described as follows.

To detect the relative copy numbers of the two *atp6* alleles, we first amplified the *atp6* gene fragment using primers ATP6H1 (5′ TGTAGCTAGAGCTTTCTCTTTAGGAGT 3′) and ATP6H2 (5′CCACTTGTAAATAATTTAGTTAAAAGACC3′). Similarly, the nuclear reference gene β-tubulin was amplified using primers TUB1 (5′CAGGAGGGTATGGATGAG3′) and TUB2 (5′TAACTGGAGGGGAGAATG3′). The amplified products containing the DNA targets were excised from 1% agarose gel under UV illumination and purified, then confirmed by sequencing to ensure the unique polymorphism is included in each *atp6* products. Furthermore, the concentration of the amplified products was measured with a spectrophotometer, using the average molecular weight of the product and Avogadro’s constant. The copy number per unit volume was then calculated by comparing to a standard curve.

To establish the standard curve for gene copy number in a sample, both the *tub* and *atp6* genes were first cloned separately using the PMD18-T cloning vector kit (CAT. 6011, TaKaRa, JAPAN) according to the manufacturer’s instructions. The recombinant vector was transformed into competent *Escherichia coli* cells and 25 μL of transformed culture was spread onto LB plates containing ampicillin (75 μg/ml) and X-gal/IPTG (CAT. R1171, ThermoFisher, United States). Transformed (white) colonies were picked and processed for plasmid isolation. Plasmid purification was done using a Plasmid Mini kit (CAT. TP01100, GENERAL BIOSYSTEMS, China). The presence of the insert in the recombinant clones was confirmed by restriction digestion. The cloned circular plasmid was quantified using a spectrophotometer and linearized with restriction enzyme *Hind* III (CAT.ER0501, ThermoFisher, United States). The stock solutions of linearized plasmid DNAs were serially diluted to obtain a standard series from 10^9^ to 10 copies per μL with each step differing by 10 folds. Then, standard curves were built using 6 serial dilutions of each of the three plasmid DNA containing three different DNA fragments, *atp6*-TT, *atp6*-CA and *tub*. For AQ-PCR, the corresponding standards series was run under the same conditions and the copy numbers of each gene in each specimen was calculated using the following formula (Godornes et al., 2007):

$$\frac{6.22\times 10^{23}\left(\frac{\mathrm{molecules}}{\mathrm{mole}}\right)\times \mathrm{DNA concentrations}\left(\frac{g}{\mu 1}\right)}{\mathrm{Number of bases pairs}\times 660\;\mathrm{daltons}}$$

6.022 × 10^23^ (molecules/mole) Avogadro’s number

660 Da: the average weight of a single base pair.

After the standard curves are established, we used quantitative-PCR (qPCR) to determines the actual copy numbers of target genes by relating the Ct values of each gene in each specimen to the standard curves (Xue et al., 2014). Each qPCR reaction was performed three times using the CFX96^®^ system (Bio-Rad, United States) that contained the AceQ qPCR TaqMan Master Mix (without ROX) (CAT.Q112, Vazyme, China), 10 uM of dual labeled hydrolysis probe (Probe-TT: FAM-TTTAGTTGCTGGTCACACATTACT-MGB for the TT combination at nucleotide sites 101 and 109 of *atp6*, probe-CA: FAM-TCTAGTTGCAGGTCATACATTATT-MGB for the CA combination at nucleotide sites 101 and 109 of *atp6*, and probe-TUB:FAM-AGCACATACACGGCATCCTGG-BHQ1 at *tub* locus), and 10 uM each of forward and reverse primers. During each round of PCR, the target and reference sequences were simultaneously amplified by AmpliTaq^®^ Gold DNA Polymerase. This enzyme has a 5′ nuclease activity that cleaves probes that are hybridized to each amplicon sequence. When an oligonucleotide probe is cleaved by the AmpliTaq Gold DNA Polymerase 5′ nuclease activity, the quencher is separated from the reporter dye, and thus increasing the fluorescence of the reporter dye. Accumulation of PCR products can be detected in real time by monitoring the increase in fluorescence of each reporter dye at each PCR cycle. Ct, slope, PCR efficiency, correlation coefficient (R2) and percentage of variance in copy numbers were calculated by using the default settings of Bio-Rad CFX Manager Version 3.1 (Bio-Rad, United States).

### Data Analysis

#### Species Identification

Because of the relatively high heterogeneity of the primary fungal DNA barcode ITS within individual samples of *C. cibarius* and related species, high quality ITS sequences are often not available for species identification within this species complex (Buyck et al., 2020; Zhang et al., 2021). Instead, as in previous studies (Buyck et al., 2011; Buyck and Hofstetter, 2011), the *tef-1* gene was used as the DNA barcode to determine the taxonomic placements of our *Cantharellus* specimens. However, at present, a *tef-1* sequence-based new species identification system is not yet available. Because the systematics and taxonomy of Asian *Cantharellus* remains largely unresolved, for this study, we chose *tef-1* sequence variations among samples within each of several closely related known species to establish a cutoff value for defining putative phylogenetic species limits.

To establish the cutoff value, we first retrieved all known *tef-1* sequences of *C. cibarius* and related species from GenBank. The following 13 known species within *C. cibarius* and related species were found to contain *tef-1* sequences from three or more specimens each: *C. anzutake*, *C. tabernensis, C. appalachiensis, C. tenuithrix, C. ferruginascens, C. cinnabarinus, C. cibarius s.s., C. tuberculosporus, C.texensis, C.decolorans, C. amethysteus, C. lateritius*, and *C. lewisii*. The distributions of these eight species have been well-documented and their morphological features and phylogenetic placements are known (Buyck et al., 2014). These sequences were used to identify the range of intraspecific *tef-1* sequence variation and establish a conservative sequence-based cutoff value for putative phylogenetic species identification within the yellow chanterelles. Here, the highest intraspecific variation within these eight species was chosen as our cutoff value to define species limits. Subsequently, *tef-1* sequence variations between our samples and those published previously for the known species of the yellow chanterelles were compared to the cutoff value. Specifically, we consider any specimen with a greater divergence than the cutoff from all known species and from each other in our sample as a potential new phylogenetic species. In contrast, specimens with *tef-1* sequence divergence lower than the cutoff value were considered as belonging to the same species.

For each of our specimens, DNA sequences obtained from both forward and reverse primers of the *tef-1* gene were assembled using the SeqMan sequence analysis software (DNASTAR, Inc.). All *tef-1* sequences obtained for our specimens and those from GenBank representing the diversity of species within the yellow chanterelles were aligned by using MAFFT 6.0 (Misawa et al., 2002) and checked manually by BioEdit 7.0.9 (Hall, 1999). Ambiguous positions at the two ends of each gene fragment were excluded from the analysis. For the *tef-1* dataset, maximum likelihood (ML) and Bayesian inference (BI) analyses were performed using RAxML 7.2.6 (Stamatakis, 2006) and MrBAYES3.1.2 (Huelsenbeck and Ronquist, 2001). The best partition schemes and evolutionary models were selected using MrModeltest v2.3. ML analyses were run with all parameters set to the default settings. GTR+G was used as the most appropriate model and the bootstrap analysis run with 1,000 replicates. BI analysis consisted of four simultaneous Markov chain Monte Carlo (MCMC) chains was run over 5 × 10^6^ generations with trees sampled every 100 generations when the average standard deviation of split frequencies was lower than 0.01. By omitting the first 25% of trees as burn-ins using the “sump” and “sumt” commands, a majority rule consensus tree was generated. The percentage of sequence divergence for each specimen from all other specimens and known species were calculated and used to define the taxonomic placements of each of our specimens.

#### Haplotype Inference of *tef-1* and *atp6* Genotype Distribution

In this study, like most other mushrooms, we consider each individual mushroom as derived from a mating event between two genetically different homokaryons. Thus, each mushroom is a heterokaryon, with each cell having two different haploid nuclei and each locus having two alleles. In the case of single nucleotide polymorphisms (SNPs), each nucleotide site in each individual specimen may be homozygous for a specific nucleotide or heterozygous, containing two different nucleotides. In the sequenced *tef-1* fragment, there are multiple SNP sites within many specimens. To better infer the relationships among specimens, we also inferred the *tef-1* haplotypes for each specimen using the Bayesian method implemented in the program PHASE 2.1 (Stephens and Donnelly, 2003). The inferred haplotype sequences were then imported into MrBAYES 3.2 to analyze the relationships among alleles from within the same and different mushroom fruiting bodies, the*atp6* sequence of each sample was assigned to one of the five *atp6* genotypes at the two polymorphic nucleotide sites (positions 101 and 109): heterozygous genotype with both CA and TT or homozygous genotypes CA, TA, CT, or TT. For specimens containing haplotypes with ambiguous phylogenetic placements, we also cloned their *tef-1* alleles and sequenced each cloned allele separately, following the protocol described for obtaining pure sequences of *atp6* and *tub*. The generated phylogenetic relationships were analyzed together with their geographic origins to identify geographic patterns of (putative) species distributions.

## Results

In this study, we analyzed a total of 363 mushroom specimens of the yellow chanterelles from 30 geographic locations in six countries. 24 of the locations were in Yunnan province in China while the remaining six were in Europe. The six European geographic locations had at least 10 specimens in each, with a range of 10 to 65. Among the 24 geographic locations from Yunnan, the sample sizes ranged from 1 to 23; with four having only one specimen each, 12 having 2 to 9 specimens each, and eight having 10 or more specimens each (Table 1). Each of these specimens were analyzed for its nuclear *tef-1* and mitochondrial *atp6* sequences. Below we describe the patterns of sequence variation among these specimens, with an emphasis on mitochondrial DNA polymorphisms.

### *tef-1* Genotyping of the 363 Yellow Chanterelle Specimens

Our sequence analyses identified that out of the 303 aligned nucleotide sites among the 363 *tef-1* sequences, 123 sites were polymorphic. These 123 SNPs resolved the 363 specimens into 47 *tef-1* sequence types (STs) (Peakall and Smouse, 2012). Among the 47 STs, 24 were each shared by more than one specimen each while the remaining 23 were found only once each. The most frequent ST was sequence type16, shared by 104 specimens from seven geographic populations. The second most frequent ST was sequence type45, shared by 75 specimens from 15 geographic populations. Among the 30 geographic samples, 23 contained more than one ST each while the remaining seven had only one ST in each local population. However, four of the seven geographic samples with only one ST each had only one specimen each, with the remaining three geographic samples each having two to five specimens. Samples from two geographic locations from Jianchuan and Shizhong contained most *tef-1* STs, both including seven STs each (Table 1).

### Phylogenetic Reconstruction and Phylogenetic Species Recognition

For phylogenetic analyses, we retrieved the *tef-1* sequences of *C. cibarius* and those closely related species as revealed based on previous studies (Buyck and Hofstetter, 2011; Foltz et al., 2013; Buyck et al., 2014). Specifically, except *C. confluens* which didn’t have any *tef-1*sequence deposited in GenBank, all known species of chanterelles were included in our comparisons. In addition, *C. anzutake, C. tuberculosporus*, and *C. yunnanensis*, which were recently described as very closely related species to *C. cibarius* s.s. and some of our Yunnan samples (Ogawa et al., 2018; Shao et al., 2021), was also included in the comparisons. In total,115 *tef-1* sequences were included in our phylogenetic analyses, with 47 sequences representing the 47 unique STs found in our current samples while the remaining 68 sequences were retrieved from NCBI to represent the diverse species within or closely related to *C. cibarius* s.s.

**FIGURE 2:**
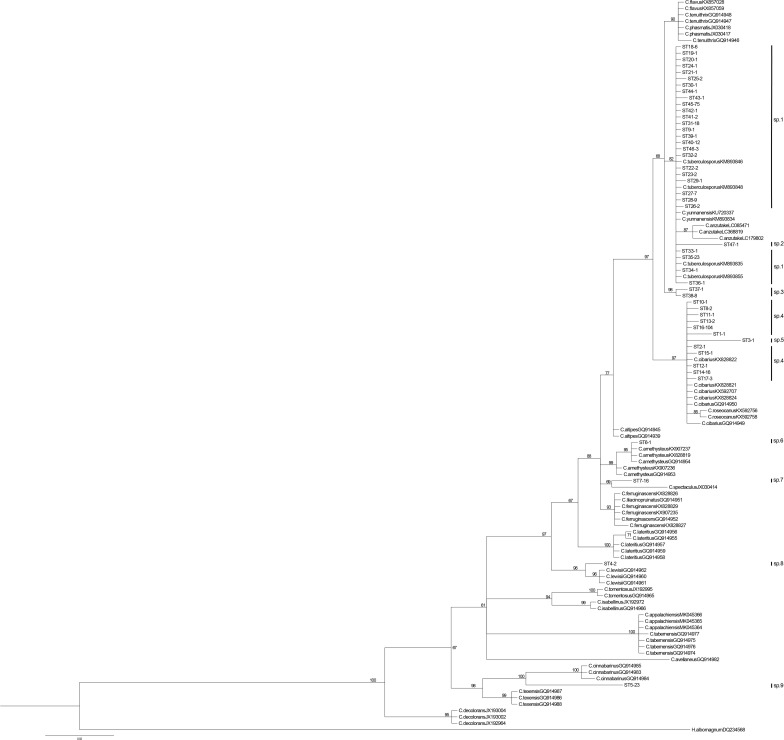
*tef-1* phylogenetic tree of the *C. cibarius* species complex. For each *tef-1* sequence type (ST) in our samples, the name or abbreviation (full name can be found in Supplementary Table 2) represents the county/community from where the sample was obtained, the number after represents the ST code assignment; the last number represents the total number of samples belonging to the specific ST.

The ML and BI analyses of *tef-1* dataset yielded identical tree topologies for the 115 *tef-1* sequences. The tree inferred from BI analysis is shown in Figure 2. As shown in Supplementary Table 1, among the 21 known reference species, *C. anzutake* showed the highest value (0.8%) of intra-specific variation of *tef-1* sequences. Thus, 0.8% sequence variation at *tef-1* locus was chosen as the cutoff value for our putative phylogenetic species identification using *tef-1* sequences within this group of chanterelles. Taking this value as a threshold, the largest interspecific genetic distances between *C. tenuithrix*, *C. phasmatis*, and *C. flavus* (0.1%) were all lower than 0.8%. Furthermore, these three species formed a single clade in our *tef-1* phylogeny. Two of these species, *C.phasmatis* and *C. flavus*, were shown to share a more recent common ancestor than with the recently described species from the southern United States *C. tenuithrix* (Foltz, 2011). Therefore, based on the 0.8% *tef-1* sequence divergence criteria to define putative phylogenetic species, *C. tenuithrix*, *C. phasmatis*, and *C. flavus* would be combined as one species/species complex. Similarly, genetic distance between *C. ferruginascens* and *C. lilacinopruinatus* was 0.1%. Indeed, both *C. ferruginaseens* and *C. lilacinopruinatus* are characterized by having a white stipe with similar pileus margin and hymenophore (Olariaga and Salcedo, 2008). Thus, these two species could be combined as one species too. The same applies to *C. roseocanus* and *C. cibarius*, which were recently separated from each other as distinct species (Foltz, 2011; Thorn et al., 2017). Thus, our criterion of requiring greater than 0.8% sequence divergence at the *tef-1* locus represents a conservative cutoff value that minimizes the number of potential new species in our collection. Based on this conservative criterion, there are likely nine putative species in our samples. Among these nine putative species, two (*Cantharellus* sp.1 and *Cantharellus* sp. 4) clustered with known species *C. tuberculosporus* and *C. cibarius*, respectively. Two others, *Cantharellus* sp. 6 and *Cantharellus* sp. 8, have <0.8% of genetic distance with the phylogenetically related species *C. amethysteus* and *C. lewisii*, respectively. The remaining five had genetic divergences from known species at a much higher than the 0.8% cutoff value, thus likely representing new putative phylogenetic species (Figure 2, Supplementary Tables 1, 2). Among the proposed new putative species, two (*Cantharellus* sp. 2 and 5) were only based on one sample each, while the other three were each based on more than one samples. Thus, to formally establish these putative species as new species, more samples need to be collected and analyzed, especially for sp. 2 and 5, in their natural habitats. Regardless of the final number of species, these results suggest a significant number of undescribed species and genotypic diversity in our analyzed yellow chanterelles.

### Evidence for Heteroplasmy Within Mitochondrial *atp6*

We successfully obtained *atp6* sequences from 327 of the 363 specimens. When we examined the *atp6* sequence chromatographs, two specimens, DL7-6 and LF-13 (both belonging to *Cantharellus* sp. 9), were found to have double peaks at sites 101 and 109, while another specimen SY6-4 had double peaks at sites 62, 95, and 132, consistent with heterozygosity at these sites. At all sites, the lower peaks were significantly higher than the baseline noise levels at neighboring homozygous sites within the chromatograph (Figure 1A). To exclude the possibility of cross-contamination, we re-sampled DNA from additional tissues of these three specimens and amplified and sequenced the *atp6* gene fragment again to confirm. For controls, genomic DNA from two other samples (ZD12-13 and YM3-30, both are in *Cantharellus* sp. 1) that initially did not show any double peaks in their *atp6* chromatographs were also re-extracted and re-sequenced. Our sequence analyses confirmed the double peaks at the same nucleotide sites in the additional tissues of DL7-6, LF-13, and SY6-4, but no evidence of heterozygosity was found in specimens ZD12-13 and YM3-30. Our results excluded the possibility of sample contamination as a cause for the observed double peaks in specimens DL7-6, LF-13, and SY6-4. In both the original chromatographs and the new chromatographs, the remaining 301 nucleotides of the sequenced *atp6* gene fragment each had only one nucleotide in specimens DL7-6, LF-13, and SY6-4, with no evidence of heterozygosity. Below, we systematically analyzed the distribution and variation patterns of heterozygosity at these sites (101 and 109). The three heterozygous sites found in specimen SY6-4 were separated from sites 101 and 109 in the following phylogenetic analyses of *atp6* gene sequences.

Comparisons using BLAST showed that both nucleotide sequences with the higher and lower peaks were most similar to the *atp6* gene from *Cantharellus cibarius* sample MG75 (GenBank number: QOWL00000000.1). The sequence identity of the *atp6* haplotype with the higher peaks to *atp6* gene of sample MG75 was 94% (E-value is 5e-099), and that with the lower peaks was 95% (E-value is 1e-101). The results are consistent with both sequences (one has the combination of the high peaks TT and the other has the combination of low peaks CA) are homologous sequences from the mitochondrial genome.

Our copy number analyses confirmed that both the TT allele and the CA allele of the *atp6* gene are more numerous than the nuclear *tub* gene in both specimens DL7-6 and LF-13. Figure 1B summarized the copy numbers of the CA and TT haplotypes and of the nuclear *tub* gene from specimens DL7-6 and LF-13. Specifically, in specimen DL7-6, the CA and TT allele copy numbers were 10.9- and 69.9-fold higher than *tub*. Similarly, in specimen LF-13, the CA and TT allele copy numbers were significantly higher than that of *tub* gene, at 2.7- and 57.1- folds of *tub*. The differential ratios of the two alleles of *atp6* gene both within each specimen and between the two specimens are also consistent with their non-nuclear nature (Sandor et al., 2018).

### Distribution of atp6 Genotypes Along the *tef-1* Phylogeny

**FIGURE 3:**
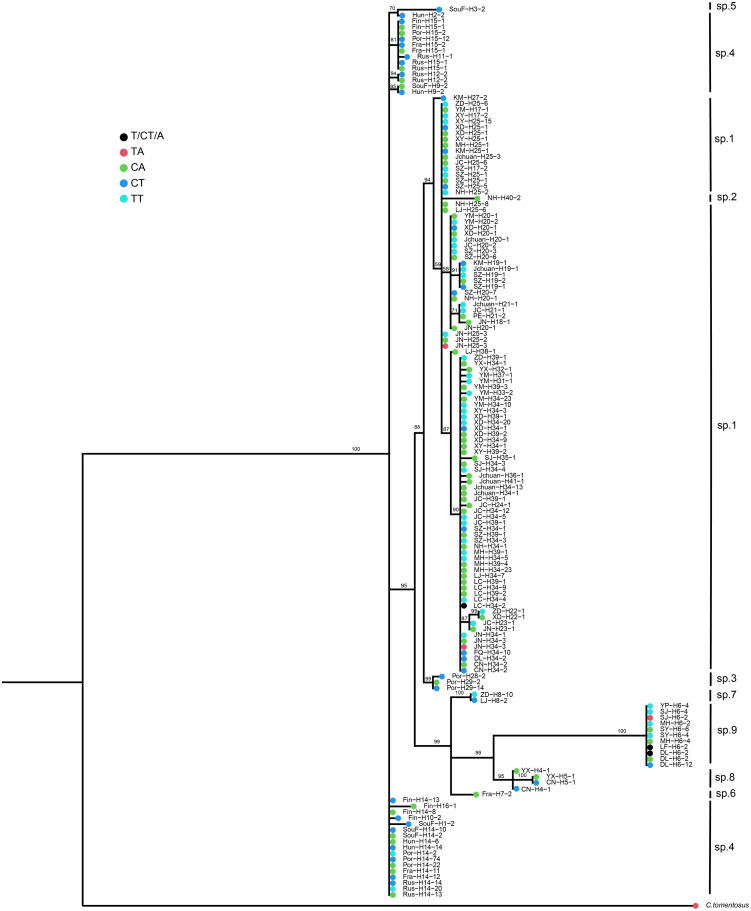
Bayesian *tef-1* haplotype cladogram showing the broad distributions of*atp6* genotypes at two polymorphic nucleotide sites. Termina branch colors represent the different *atp6*genotypes at the two polymorphic sites; For each *tef-1* haplotype in our samples, the name or abbreviation (full name can be found in Supplementary Table 2) represents the county/community from where the sample was obtained, the number after “H” represent the haplotype assignment; the last number represents the total number of samples belonging to the specific haplotype from each geographic location and for each unique *atp6* genotype. Only representative sequences of unique haplotypes from each geographic location and unique *atp6* genotypes are shown.

The phylogenetic distributions of *atp6* haplotypes at the two nucleotide sites (sites 101 and 109) were mapped onto the Bayesian tree constructed based on the *tef-1* dataset (Figure 3). The results showed that the four different haplotype combinations (CA, CT, TA, and TT) at these two sites were broadly distributed throughout the *tef-1* sequence-based phylogenetic tree. For example, among the European samples of the yellow chanterelles, three haplotypes CA, CT, and TT were found at these two nucleotide sites. Similarly, in multiple clades of specimens from Yunnan, three to four haplotypes out of all four possible haplotypes were found at these two *atp6* nucleotide sites. Specifically, except for two clades *Cantharellus* sp. 2 and *Cantharellus* sp. 5 (both with just one sample each, Figure 2), other seven putative phylogenetic species recognized by *tef-1* sequences have two or more different *atp6* haplotype combinations. Within *Cantharellus* sp. 1 and *Cantharellus* sp. 9, all four haplotypes at the two sites were found, consistent with recombination within this mitochondrial gene fragment in the population. However, if true, during subsequent evolution and diversification, multiple parallel mutations and/or reversions at the two sites must have occurred to account for the observed patterns of distribution. However, given the low sequence diversity among the samples at *atp6* gene fragment, we believe that the chance of parallel mutation or reversal at exactly these two sites in multiple clades is extremely small. The second possibility is that mitochondrial recombination is frequent within and among contemporary populations of the yellow chanterelles throughout their distribution range, a single recombination event from the heteroplasmic parent followed by segregation could have generated all four possible genotype combinations at these two sites. However, this second possibility requires most of the proposed species to hybridize with each other to generate recombinant mitochondrial genotypes. The third possibility is that the ancestor of the yellow chanterelles was heteroplasmic and recombined to produce four haplotypes between these two polymorphic nucleotide sites. These recombinants and heteroplasmic individuals then diversified among descendent lineages but with incomplete lineage sorting, resulting in some lineages maintaining the heteroplasmic state while others containing one to four of the four possible haplotypes.

**FIGURE 4:**
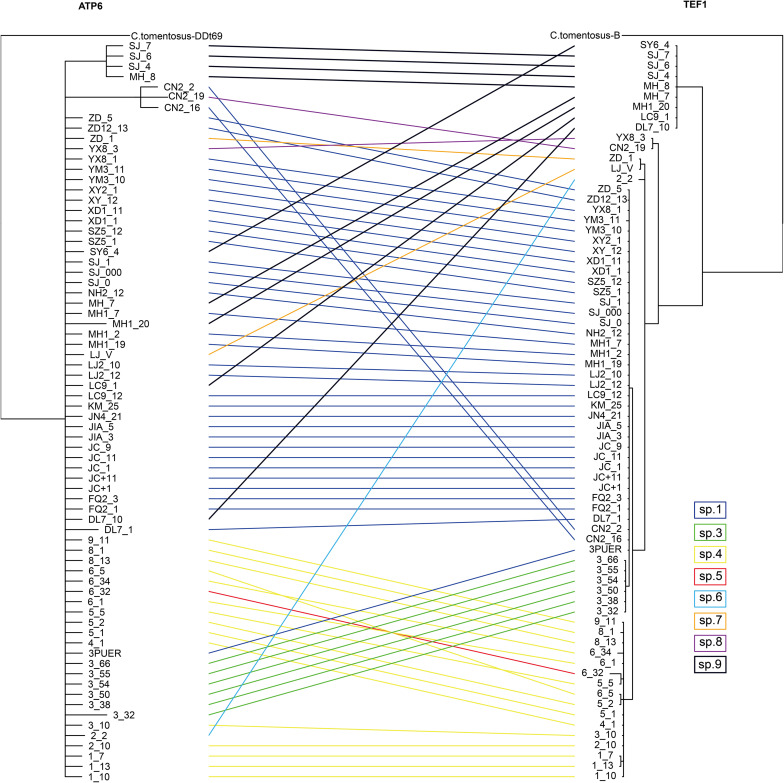
The tanglegram between *tef-1* and *atp6* phylogenies. The full name can be found in Supplementary Table 2.

To further investigate evidence of recombination among polymorphic nucleotide sites within the mitochondrial *atp6* gene, we measured the degree of association between alleles at seven polymorphic nucleotide sites within this DNA fragment using the program Multilocus 1.3 (Agapow and Burt, 2001). The analyses were conducted for both the total samples and samples from individual phylogenetically distinct clusters as revealed by the *tef-1* phylogeny. Only clades with >5 samples were analyzed. Because I_*A*_ can be influenced by the number of polymorphic loci and samples, we standardized the I_*A*_ value by the rBarD value for comparisons among populations. The null hypothesis for I_*A*_ is that there is random association (recombination) among alleles at different loci, a p value of <0.05 would indicate that the null hypothesis should be rejected. Phylogenetic incompatibility (PrC) is another indicator of recombination at the population level, the lack of phylogenetic incompatibility implies no recombination (Agapow and Burt, 2001). Results of I_*A*_ and PrC tests showed that while random recombination was rejected in all samples, evidence for recombination was found in both the total sample as well as in *Cantharellus* sp. 9 and *C. tuberculosporus* (Table 2). Specifically, they showed clear evidence of phylogenetic incompatibility (Table 2). Overall, our results are consistent with non-random recombination in the mitochondrial genome of several distinct clades within the yellow chanterelles.

**TABLE 2:** Association among SNPs at the *atp6* gene for *C. cibarius* and related species across southwestern China and Europe.

Species groups	Sample size	Phylogenetic compatibility (p value)	rBarD (p value)
All samples	327	0.7826 (< 0.001)	0.1827 (< 0.001)
*Cantharellus* sp. 3	9	1 (0.035)	1 (0.035)
*C. tuberculosporus*^1^	158	0.9710 (< 0.001)	0.3004 (< 0.001)
*Cantharellus* sp. 7	5	1 (1)	n/a^3^
*Cantharellus* sp. 9	14	0.8986 (< 0.001)	0.3488 (< 0.001)
*C. cibarius*^2^	132	1(1)	0.3726 (< 0.001)

*1. Known species from Yunnan; 2. Known species from Europe; 3. Not analyzed due to small sample size.*

Using the same seven non-heteroplasmic sites within the *atp6* gene fragment, we constructed the phylogenetic relationships of all clone-corrected samples and compared it with that constructed using *tef-1* SNPs. The phylogenetic comparisons revealed evidence for phylogenetic in congruences between the two gene trees among most phylogenetic species. An example is strain SY6-4 that showed distinctly incongruent phylogenetic placements between the *tef-1* and *atp6* phylogenies. The results further support recombination and hybridization within and between several clades within the yellow chanterelles.

## Discussion

### Species and Genetic Diversity

In this study, 363 fruiting bodies of the edible yellow chanterelles were obtained from Yunnan, Southwestern China and five countries in Europe. Phylogenetic analyses of our *tef-1* sequence types and closely related sequences from GenBank identified that while many of our samples belonged to two known species *C. cibarius* (most of our European samples) and *C. tuberculosporus* (many of our Yunnan samples), our specimens belonged to seven additional putative phylogenetic species.

Previous studies have shown that the broad application of the name *C. cibarius* to chanterelles found in North America and other areas outside of Europe were inaccurate. As a result, several new species have been described (Buyck and Hofstetter, 2011). The phylogenetic analyses of our samples from Southwest China along with representative samples of *C. cibarius* and related species from other parts of world indicated that the Yunnan and European samples contained a total of nine putative phylogenetic species. However, due to the lack of ITS, *tef-1*, and other gene sequences in public databases for several known species in this group of mushrooms, we are unable to confidently finalize the phylogenetic species status of the four clades. Indeed, though ITS shows higher resolution for species identification after cloning step (Ogawa et al., 2018), the difficulties in the direct amplification and sequencing of this gene, as well as the shortage of ITS data in the public database due to its high frequency of heterogeneity in many *Cantharellus* samples, have made ITS not an ideal barcode locus for this group of fungi (Xu, 2016). Instead, *tef-1* has been recognized as the DNA barcode for *C. cibarius* and related species (Olariaga et al., 2017).

By constructing the *tef-1* phylogenetic tree with the reference sequences representing the few current known species within the edible yellow chanterelles, we made several novel observations. First, several known species distinguished based on morphological characters showed no or very limited difference between each other at the *tef-1* gene fragment. Indeed, the lack of a clear barcode gap at the *tef-1* gene among several groups of morphological species suggest that additional genes or whole-genome sequences should be used to confirm the genetic uniqueness of these species (Xu, 2020). Second, most of the samples from Yunnan had distinct *tef-1* sequences from those from other parts of the world. For example, most of the samples from Yunnan belonged to one clade that clustered with *C. anzutake, C. tuberculosporus*, and *C. yunnanensis* (Ogawa et al., 2018; Shao et al., 2021; Zhang et al., 2021). However, until recently, the common chanterelles in Yunnan province were called *C. cibarius*, the species found only in Europe (Liu et al., 2009; Wang et al., 2009). The known species *C. tuberculosporus* and *C. yunnanensis* showed very low interspecies genetic distance (0.3%) from each other, and with only 0.8% between *C. yunnanensis* and *C. anzutake*. According to the cutoff value we established for species delimitation in this study, the three species could be combined into one monophyletic species. Thus, our analyses indicate that the name *C. cibarius* is a misnomer when applied based on morphological characteristics to chanterelles in Yunnan, a conclusion similar to what was found recently through morphological investigations (Shao et al., 2021). With such a stringent criterion, several specimens from Zhongdian (*Cantharellus* sp. 7) and Nanhua (*Cantharellus* sp. 2) in Yunnan most likely belonged to novel species. Further comparisons using morphological characteristics and additional DNA sequences are needed to confirm their genetic uniqueness and cryptic speciation.

### Persistent Heteroplasmy and Ancient Mitochondrial Recombination

We found evidence for heteroplasmy and recombination within the mitochondrial gene *atp6* in our samples of *C. cibarius* and related species. The identification of heterozygous nucleotide sites at the *atp6* locus in samples DL7-6 and LF-13 is consistent with heteroplasmy. In laboratory crosses, heteroplasmy is a pre-requisite for mitochondrial recombination (Basse, 2010). Furthermore, according to the *tef-1* phylogeny, although the two heteroplasmic specimens DL7-6 and LF-13 belonged to the same *tef-1* clade, the four *atp6* haplotypes as observed from the yellow chanterelles had wide geographic and phylogenetic distributions, indicating that recombination was likely ancient. For recombination to occur, heteroplasmy must have been present in the ancestor to drive the recombination event. However, during subsequent evolution and diversification, most lineages may have lost heteroplasmy and/or some of the recombinant *atp6* genotypes. The exceptions are two specimens DL7-6 and LF-13 that maintained their heteroplasmy.

Advances in DNA sequencing and polymorphism detection technology have documented the presence of recombinant mtDNA for a wide range of eukaryotic taxa. In the Baker’s yeast *Saccharomyces cerevisiae*, there is a large body of data showing evidence of heteroplasmy and recombination in laboratory crosses. However, mitochondrial heterogeneity was always transient, with the offspring of the zygote becoming completely homozygous within 20 mitotic cell divisions (Hermann and Shaw, 1998). A few other studies have also provided evidence of mtDNA recombination in natural populations of certain fungal species, such as *Armillaria gallica* (Saville et al., 1998), *Cryptococcus gattii* (Xu et al., 2009), *Agaricus bisporus* (Xu et al., 2013) and recently, *Thelephora ganbajun* (Wang et al., 2017). However, there has been no report showing evidence of potential stable heteroplasmy and broad mitochondrial recombination over repeated speciation events in nature.

In the human pathogenic yeast *C. neoformans*, a progeny population with diverse mitochondrial genotypes created due to biparental mitochondrial inheritance was found to show greater phenotypic variation than that with a uniparental mitochondrial inheritance (Yan et al., 2007). Mitochondrial genomes are commonly inherited uniparentally in the majority of sexual eukaryotes. As a result, these genomes are considered effectively asexual that may be prone to Muller’s Rachet, an irreversible accumulation of deleterious mutations (Lynch, 1996; Xu, 2005). Interestingly, our analyses identified signatures of heteroplasmy and mtDNA recombination in the yellow chanterelles, suggesting these organisms may have an effective mechanism to prevent Muller’s Rachet from operating in their mitochondrial genomes (Matsumoto and Fukumasa-Nakai, 1996; Xu, 2007).

At present, the selective pressure responsible for the maintenance of heteroplasmy and mitochondrial recombination in the yellow chanterelles is not known. In the human pathogenic yeast *C. neoformans*, high temperatures and UV exposure have shown to be capable of changing mitochondrial inheritance from uniparental to biparental (Yan et al., 2007), which also included mitochondrial heteroplasmy and the generation of mitochondrial recombinants. The high altitudes in Yunnan where these mushroom samples are from may be exposed to significant UV irradiations or other stress factors that favor mitochondrial heteroplasmy and recombination as an adaptive response to reduce the rate of accumulation of deleterious mutations in the mitochondrial genomes in these species. Indeed, evidence for mitochondrial recombination have been found in several other mushroom species (e.g., *T. ganbajun* and a *Russula virescens* ally) in the same geographic regions (Cao et al., 2013; Wang et al., 2017). Further investigations of the relationships between environmental factors and mitochondrial genetic variations are needed to determine the extent of mitochondrial recombination and the potential adaptive significance of such recombination in these mushrooms.

### Comparison Between Mitochondrial and Nuclear Genetic Polymorphisms

In our analyses, there were overall lower sequence divergences among clades at the *atp6* locus than those at *tef-1* locus. This result is consistent with what has been reported in many other fungi that showed lower rates of evolution in the mitochondrial genome than that in the nuclear genome (Sandor et al., 2018, Sandor et al., 2020). Indeed, distinct genotypes and lineages identified based on the nuclear *tef-1* sequences were often found to share the same *atp6* sequences. However, the lower sequence diversity for mitochondrial genes than nuclear genes in these mushrooms doesn’t mean that the mitochondrial genomes in these fungi are static. Contrary, the mitochondrial genome size variation among strains in the same or closely related species can be very large (up to ±25% of the mean genome size), usually much greater than the nuclear genome size variation within many fungal species (Sandor et al., 2018; Wang and Xu, 2020; Zhang et al., 2020). The mitochondrial genome size variations in fungi were mainly due to differences in the number and size of introns and mobile genetic elements (Sandor et al., 2018; Wang and Xu, 2020; Zhang et al., 2020). Indeed, in our initial screening for mitochondrial genetic markers for analyzing the yellow chanterelles, polymorphisms in intron distributions were found in several genes, including *cox1, cox2, cox3, nad1, nad5, cob*, and *rnl* (Supplementary Table 3 and unpublished data). Unfortunately, such intron distribution polymorphisms in these genes limited the success of our direct PCR and sequencing efforts for these specimens. Consequently, these gene fragments were not selected as markers to further investigate the mitochondrial genetic polymorphisms in our study.

We would like to mention that while the low rate of evolution at the mitochondrial *atp6* gene may be responsible for some of the haplotype sharing, an alternative explanation is recent hybridization among some of the lineages. Indeed, our analyses revealed evidence of incongruence between the nuclear *tef-1* phylogeny and the mitochondrial *atp6* phylogeny, consistent with hybridization among lineages. Analyses of whole-genome sequences, including both the nuclear and mitochondrial genome sequences at the population level, is needed in order to distinguish these two possibilities.

## Data Availability Statement

The data presented in the study are deposited in the GenBank database, accession number(s) can be found in Supplementary Table 2.

## Author Contributions

YZ and JX: Conceptualization, validation, visualization, supervision, project administration, and funding acquisition. JX, SW, and MM: sample collection. SW, HL, CL, FM, RW, and MM: methodology. SW and HL: software and formal analysis. YZ, JX, and SW: investigation. YZ and SW: writing—original draft preparation. JX: writing—review and editing. All authors have read and agreed to the published version of the manuscript.

## Conflict of Interest

The authors declare that the research was conducted in the absence of any commercial or financial relationships that could be construed as a potential conflict of interest.

## Supplementary Material

Supplementary Table 1 Average evolutionary divergence over *tef-1* sequence pairs within and between groups (provincially adopted phylogenetic species) calculated by MEGA 6 using the K2P model.

Supplementary Table 2 List of putative species, corresponding ST code and samples used in Figures 2, 4, *tef-1* haplotype code, corresponding *atp6* genotype and sample origins used in Figure 3.

Supplementary Table 3 Intron numbers of mitochondrial genes in *C. cibarius* and related species.
